# Supplementary material for: Discovering the drivers of clonal hematopoiesis
Source: Nat Commun. 2022 Jul 23;13:4267. doi: 10.1038/s41467-022-31878-0 (PMC9308779; doi:10.1038/s41467-022-31878-0)
Supplement: Supplementary file 1 — Supplementary Information [file 41467_2022_31878_MOESM1_ESM.pdf]

Supplementary Material of

Discovering the drivers of clonal hematopoiesis

Oriol Pich[1], Iker Reyes-Salazar[1], Abel Gonzalez-Perez[1,2,^], Nuria Lopez-Bigas[1,2,3,^]

1. Institute for Research in Biomedicine (IRB Barcelona), The Barcelona Institute of Science and Technology, Baldiri Reixac, 10, 08028 Barcelona, Spain.
2. Research Program on Biomedical Informatics, Universitat Pompeu Fabra, Barcelona, Catalonia, Spain.
3. Institució Catalana de Recerca i Estudis Avançats (ICREA), Barcelona, Spain

^Corresponding authors
Abel Gonzalez-Perez: abel.gonzalez@irbbarcelona.org
Nuria Lopez-Bigas: nuria.lopez@irbbarcelona.org

Table of Contents

Supplementary Note 1 2

References 18

Supplementary Figures 21

Supplementary Note 1

Comments on the reverse calling
The reverse calling approach implemented in this work for the identification of blood somatic mutations has two main advantages with respect to one-sample germline or somatic calling.

First, variants supported by fewer reads than the minimum generally required in a germline calling may be identified. It is this difference that accounts for the gain in sensitivity in the identification of CH across samples in the metastasis cohort exemplified in Figure 1c and Supplementary Figure 5c. This is a key feature in the aim to repurpose cancer genomics datasets for the discovery of CH driver genes, given that the blood samples across cancer patients cohorts are sequenced at lower depths than their tumor counterparts (e.g., ~40X in the metastasis cohort). Deeper sequencing of these paired blood samples across cancer genomics datasets would definitely favor this repurposing.

Second, the availability of a second sample from the same individual improves the filtering of germline variants of the reverse calling with respect to any filter of polymorphisms implemented posterior to germline calling of the blood sample in isolation. This thus determines that the reverse calling is more specific than a germline calling. In order to precisely ascertain this gain in specificity of somatic mutation calling arising from the implementation of the reverse calling, we compared the number (and VAF distribution) of variants identified through the reverse calling and the regular germline calling in the 15 well-known CH genes across the 3,785 donors in the metastasis cohort. For the purpose of this comparison, the VAF distribution of variants from the germline calling was cut at 0.5, and common polymorphisms present across databases such as dbSNP and gnomAD were removed, exactly as in the case of the reverse calling post-processing. The results (presented in Figure 1c of the main paper) show that more than 91% of the variants identified by the regular germline calling are not present in the reverse calling due to their presence in the reference (tumor) sample from the donor. The distribution of VAF of these variants (close to 0.5) supports the suspicion that they contain many germline variants. This difference highlights the gain in specificity provided by the reverse calling (i.e., exploiting the second sample from the donor's tumor as germline reference).

We next asked whether results similar to those of the reverse calling could be obtained by calling potential somatic mutations from a single blood sample, for example using the Mutect2 single sample mode. We hypothesized that the reverse calling would produce a more specific dataset of somatic mutations than the single-sample somatic calling, since germline variants are expected to be filtered in the calling process from their presence in the reference (tumor) sample. To answer this question, we recalled somatic mutations across 276 samples randomly chosen from the primary cohort using Mutect2 in the single sample mode. We then compared the distribution of VAF of the mutations identified by the reverse calling and this single-sample somatic calling (Fig. 1a Supplementary Note 1). The single-sample somatic calling approach identifies a larger number of mutations across these 276 samples, which are shifted towards higher VAF values. This means, as expected, that potentially, many of the mutations (with VAF close to 0.4) identified by the single-sample somatic calling approach are actually germline

variants filtered out in the reverse calling approach due to their presence in the reference sample.

One potential caveat of the reverse calling approach is that the VAF filter applied to the blood mutations called (VAF<0.5) may result in the loss of true blood somatic mutations that occur in genomic regions that suffer a mosaic copy number loss in the process of CH. We have assessed that the number of such lost CH cases (based on the list of known CH drivers) is minimal (see Figure 1b Supplementary Note 1), and we have decided to err on the side of caution by keeping this filter to avoid the potential inclusion of falsely called blood somatic mutations.

In order to identify other potential pitfalls of the reverse calling approach, we analyzed several non-canonical scenarios of its operation and their potential outcomes. In genomic sites that bear somatic mutations in the tumor (i.e., in which the reference allele, but also an alternate are present), if the site is not polymorphic, the most likely outcome of the reverse calling is a non mutated site. The reason is that the reference allele is present in both the query and reference sample. Nevertheless, if a mutation were called in this site, it would be eliminated by the filter of VAF implemented downstream the calling (see above and Figure 1 of the main article), as it would appear with a VAF close to 1. A more complicated scenario is posed by somatic mutations in the tumor that correspond to polymorphic sites in the genome. In this case, a false positive blood somatic mutation could be called, because one of the alleles in the query sample (blood) will differ from the two present in the reference sample (tumor). However, such false somatic mutation would be eliminated by the filter of polymorphisms implemented downstream the calling, except in the case that the variant in the germline genome of the donor is not a polymorphism, but private. We specifically quantified the case of blood somatic mutations called overlapping tumor mutations. Across all blood somatic mutations identified in the metastasis cohort (1,369,926 mutations), 99 (0.00007) overlap mutations in the tumor (with the same or different allele). Seven of these correspond to coding genes or the 64 genes identified as CH drivers (3 in *DNMT3A*, 2 in *SF3B1*, 1 in *TP53*, and 1 in *JAK2*). Interestingly, in these seven cases, exactly the same mutation (nucleotide change) is found in the blood and the tumor, suggesting that at least some of these cases might correspond to blood somatic mutations detected in the tumor due to leukocyte infiltration. We thus conclude that the reverse calling approach is robust to the presence of tumor point somatic mutations, and only in very rare occasions it may lead to a false blood somatic mutation call.

Another complex scenario corresponds to an event of loss of heterozygosity (LOH) in the tumor sample. Private germline variants of the donor overlapping such an event have the potential to be falsely called as blood somatic mutations. The majority of these variants will appear as mutations with VAF close to 0.5, and are filtered out in the mosaic set (see Figure 1c Supplementary Note 1). To avoid that genes with possible false mutation calls in LOH regions in the tumor or overlapping private variants of the donor are included in the compendium, we decided to filter out blood somatic mutations with VAF>0.4 from the sets of mutations employed in the discovery of CH driver genes.

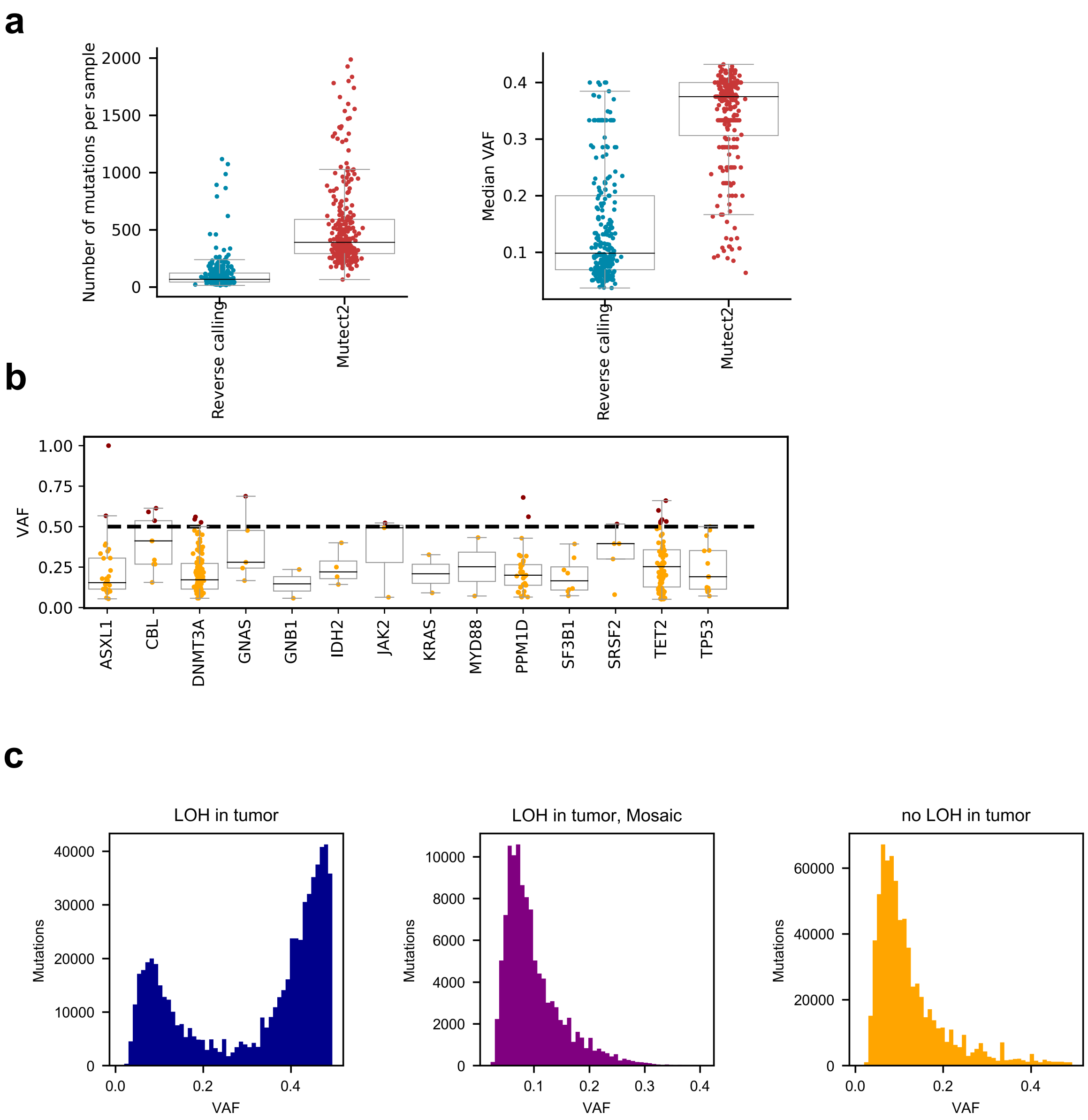

a
Number of mutations per sample
2000
1500
1000
500
0
Reverse calling
Mutect2
Median VAF
0.4
0.3
0.2
0.1
Reverse calling
Mutect2
b
VAF
1.00
0.75
0.50
0.25
0.00
ASXL1
CBL
DNMT3A
GNAS
GNB1
IDH2
JAK2
KRAS
MYD88
PPM1D
SF3B1
SRSF2
TET2
TP53
c
LOH in tumor
Mutations
40000
30000
20000
10000
0
0.0
0.2
0.4
VAF
LOH in tumor, Mosaic
Mutations
10000
8000
6000
4000
2000
0
0.1
0.2
0.3
0.4
VAF
no LOH in tumor
Mutations
60000
40000
20000
0
0.0
0.2
0.4
VAF

Figure 1 Supplementary Note 1. Potential caveats of the reverse calling approach.
a) Comparison of the results obtained with the application of the reverse calling and a single-sample somatic calling approach on 276 samples of the primary cohort. Left plot: distribution of number of variants identified by both approaches across samples; right plot: distribution of median VAF of variants identified by both approaches across samples. In the boxplots, the box represents the second and third quartiles, separated by a line indicating the median; the whiskers represent the minimum and maximum of the distribution excluding outliers.
b) Distribution of variant allele frequency of blood somatic mutations affecting known CH drivers across the metastasis cohort. The broken horizontal black line represents the threshold of VAF used as a filter. Very few mutations (in red) possess VAF greater than 0.5 are thus lost due to the application of this filter. In the boxplots, the box represents the second and third quartiles, separated by a line indicating the median; the whiskers represent the minimum and maximum of the distribution excluding outliers.
c) Distribution of VAF (variant allele frequency) of blood somatic mutations overlapping LOH in tumor (left), and non-LOH (right) regions of the genome across donors of the metastasis cohort. The plot at the center presents the distribution of VAF of blood somatic mutations overlapping LOH regions in the tumor in the mosaic set.

Identifying genes under positive selection in CH

We posit that if somatic mutations identified across healthy blood samples of patients in both cohorts are true hematopoietic mutations that become detectable due to clonal hematopoiesis, then their distribution across the genome is not expected to be completely random. Instead, because clonal hematopoiesis is a phenomenon driven by mutations that provide some hematopoietic stem cells with advantages with respect to others, signals of positive selection are expected to be detectable in their distribution across the genome. Specifically, these signals would be apparent in genes whose mutations are under positive selection in the arisal of clonal hematopoiesis. Thus, we expect that mutations in these CH-related genes exhibit distinct patterns of mutations that deviate from the expected under neutral evolution.

A range of methods to identify these signals of positive selection across genes have been developed in recent years for their application to tumor genomics data with the aim of identifying cancer driver genes. If detected, the signals of positive selection across the somatic mutations in blood samples would thus be a reflection of the clonal expansion triggered by CH.

Therefore, we applied the IntOGen-pipeline[19], which runs seven complementary state-of-the-art methods[26,35–40] to detect signals of positive selection in the mutation pattern of genes. These methods are designed to detect different deviations of the mutation pattern of genes with respect to their expectation under neutrality --that is, different signals of positive selection. The methods were applied independently to the full and mosaic (see main text) sets of somatic mutations identified across the blood samples of both datasets. As a general rule (i.e., except a couple of cases) the genes with signals of positive selection according to the different methods in the different cohorts and mutations sets are significantly enriched for known cancer driver genes --i.e., those that when mutated confer an advantage to somatic cells (CGC[41] genes in Table 1). The same significant enrichment is apparent for known drivers of clonal hematopoiesis (CH in Table 1) and genes that drive specifically myeloid malignancies (Myeloid[12] in Table 1).

We also employed quantile-quantile plots (qqplots) of the results of the different methods (in which the p-values of a set of genes deviate from the uniformity that would be expected under neutrality) to assess their calibration (Fig. 2 Supplementary Note 1). Most methods, when run on the mosaic sets of mutations derived from both cohorts exhibit a well calibrated behavior, with only a few significant genes deviating from the diagonal. We hypothesize that in the few cases in which an inflation is observed, the reason is that (unlike in the case of cancer somatic mutations) the sets of mutations employed to run the methods are still contaminated with artifactual mutations with a non-random distribution which may contribute to biasing their results.

Driver identification methods based on different signals of positive selection also show different biases when applied to cancer somatic mutations. This is why we have developed a reasoned approach to combine their outputs that delivers weights on the basis of the perceived credibility of the methods in each cohort[19]. Thus, we anticipate that the biases observed in the results of certain methods when applied to blood somatic mutations in both cohorts, could be solved using this combination approach.

Moreover, because the full set of mutations identified through the reverse calling approach may be contaminated by potential sequencing artifacts, we deem the set of clonal hematopoiesis genes identified by the combination of the results of the application of the methods to these filtered catalogs (Mutect and Mosaic) more reliable than that obtained from the full catalog.

In order to derive the list of clonal hematopoiesis drivers, we thus start with genes that are significant (in the combination of methods' outputs) from the analysis of any filtered catalog. Genes that appear significant from the analysis of the full catalog are only included in the final list if they are supported by prior knowledge of their involvement in CH or cancer (i.e., included in the CGC[41]). This way, we generate two lists of putative CH drivers, i.e., one for each cohort. These two lists, directly obtained from the combination algorithm, are already very enriched for known CH and cancer genes (Table 2). The genes in these two lists, among which false positives may still be present, are carefully vetted employing criteria that we have developed in a decade-worth of analysis of cancer cohorts (https://intogen.readthedocs.io/en/latest/postprocessing.html#). Specifically, we remove from the lists:

- 10 genes that are not expressed (their highest expression value is below 15 fragments per kilobase of transcript per million mapped reads, or fpkms) across a set of HSCs (see methods). The names of these non-expressed discarded genes are highlighted in Figure 3 Supplementary Note 1 with a "#" symbol preceding them.
- 1 gene due to be highly tolerant to Single Nucleotide Polymorphisms[7] (SNP) across human populations
- 2 genes that are frequent false positives of different driver discovery methods
- 1 gene that has more than 3 mutations in one sample, which may be a signal of a local hypermutation process or contamination of germline variants from the reverse calling
- 1 gene that has more than 50% mutations in one of the cohorts associated with COSMIC Signature 9 (https://cancer.sanger.ac.uk/cosmic/signatures), related with the maturation of lymphoid cells

The names of these 5 genes discarded due to different reasons are included in Table 2 with the label "DISCARDED".

The vetting process yields two lists composed of 23 and 33 genes, all of which are reasonably good candidates of driving clonal hematopoiesis in either cohort (Table 2). The vast majority (21/23 and 26/33) have been mentioned in the literature related to CH, myeloid malignancies, or tumorigenesis in general[21,42]. Although the genes with no prior knowledge of involvement in any of these processes could be bona fide CH genes, further evidence is needed. With the objective to generate a very reliable snapshot of the compendium, these genes are discarded from the compendium, but still listed in Table 2 of this Supplementary Note.

Primary

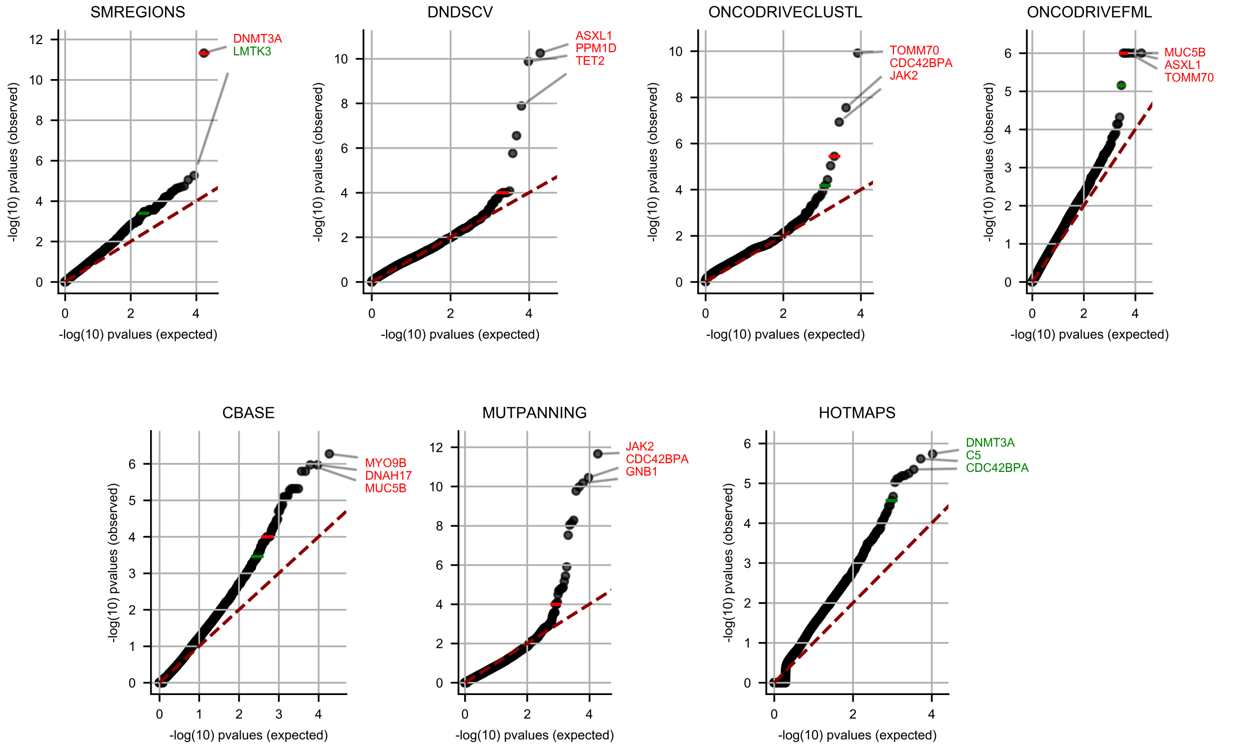

Metastasis

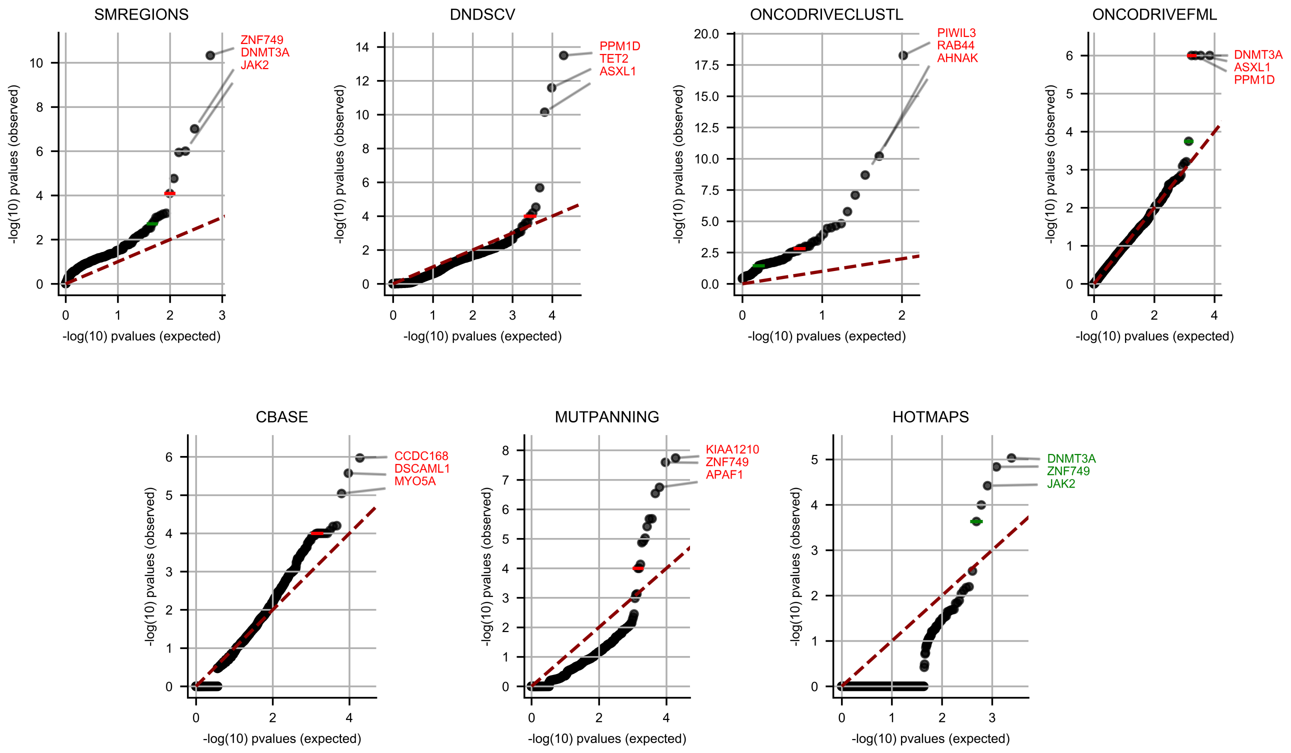

Figure 2 Supplementary Note 1. Quantile-quantile plots of driver discovery methods in the detection of CH.

QQplots of the results of analyzing the mosaic set of mutations are represented. Parametric, non-parametric or empirical statistical tests implemented by the seven methods in the IntOGen pipeline are described in their original articles. The names of genes that are significant at FDR 0.01, appear in red, while those significant at FDR 0.1, appear in green.

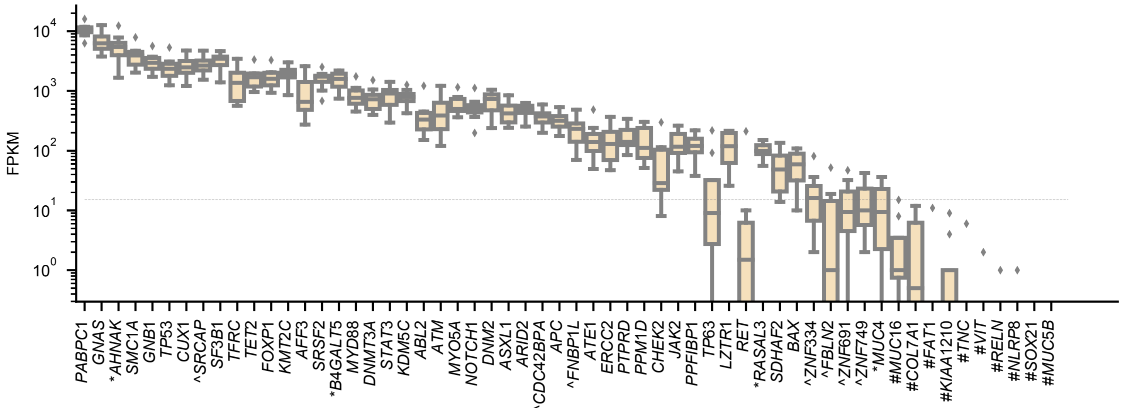

FPKM
10^4
10^3
10^2
10^1
10^0
PABPC1
GNAS
*AHNAK
SMC1A
GNB1
TP53
CUX1
^SRCAP
SF3B1
TFRC
TET2
FOXP1
KMT2C
AFF3
SRSF2
*B4GALT5
MYD88
DNMT3A
STAT3
KDM5C
ABL2
ATM
MYO5A
NOTCH1
DNM2
ASXL1
ARID2
^CDC42BPA
APC
^FNBP1L
ATE1
ERCC2
PTPRD
PPM1D
CHEK2
JAK2
PPFIBP1
TP63
LZTR1
RET
*RASAL3
SDHAF2
BAX
^ZNF334
^FBLN2
^ZNF691
^ZNF749
*MUC4
#MUC16
#COL7A1
#FAT1
#KIAA1210
#TNC
#VIT
#RELN
#NLRP8
#SOX21
#MUC5B

Figure 3 Supplementary Note 1. Expression of genes under positive selection in the primary and metastasis cohorts across HSCs.

Most genes in the list of putative CH drivers identified through the IntOGen pipeline appear expressed across HSCs cells (i.e., above the threshold of 15 fpkm), while only 9 that appear below this value are filtered out. Genes that are filtered out due to lack of expression are marked with '#'; genes that are filtered out due to other criteria (detailed above) bear the '*' label; genes discarded due to lack of literature evidence of involvement in CH or tumorigenesis are labeled '^'. The gene *TOMM70*, not included in the expression dataset employed for this purpose (see Methods of the main paper) is also excluded from the list of potential CH driver genes. In the boxplots, the box represents the second and third quartiles, separated by a line indicating the median; the whiskers represent the minimum and maximum of the distribution excluding outliers.

Table 1 Supplementary Note 1. Enrichment of different discovery methods applied to different sets of mutations called in the primary and metastasis cohorts on known CH, Myeloid and cancer genes. Odds ratios and p-values correspond to one-tailed Fisher's exact test.

Cohort	Mutations set	Method	Fraction of known genes			Total genes			Odds ratio			P-value		
			CH	Myeloid	CGC	Identified by the method	In no list	In any list	CH	Myeloid	CGC	CH	Myeloid	CGC
Metastasis	Mutect	Cbase	0.80	0.80	0.80	5	1	4	6781.8	816.3	103.1	1.3E-12	3.1E-09	9.6E-06
Metastasis	Mosaic	Cbase	0.44	0.44	0.44	9	5	4	1356.1	163.2	20.6	3.4E-11	7.8E-08	2.1E-04
Metastasis	Full	Cbase	0.57	0.57	0.57	7	3	4	2260.4	272.1	34.4	9.4E-12	2.2E-08	6.3E-05
Primary	Mosaic	Cbase	0.21	0.21	0.21	19	15	4	451.8	54.4	6.9	1.0E-09	2.3E-06	4.9E-03
Primary	Full	Cbase	0.20	0.20	0.25	20	15	5	423.5	51.0	8.6	1.3E-09	2.9E-06	7.1E-04
Metastasis	Mutect	OncodriveCLUSTL	1.00	1.00	1.00	2	0	2	inf	inf	inf	0.11	0.11	0.11
Metastasis	Mosaic	OncodriveCLUSTL	0.15	0.15	0.15	20	17	3	7.2	14.6	4.8	0.05	0.02	0.08
Metastasis	Full	OncodriveCLUSTL	0.02	0.03	0.05	144	137	7	1.4	1.1	0.6	0.70	0.76	0.20
Primary	Mosaic	OncodriveCLUSTL	0.25	0.25	0.25	4	3	1	251.6	51.0	6.6	5.8E-03	0.03	0.18
Primary	Full	OncodriveCLUSTL	0.03	0.03	0.08	59	54	5	42.2	5.7	2.0	1.5E-03	0.05	0.19
Metastasis	Mosaic	SMRegions	0.33	0.33	0.33	6	4	2	53.2	19.6	4.7	2.7E-03	0.01	0.11
Metastasis	Mutect	SMRegions	1.00	1.00	1.00	1	0	1	inf	inf	inf	0.08	0.10	0.23
Metastasis	Full	SMRegions	0.07	0.07	0.07	42	38	4	17.2	5.3	0.9	2.2E-03	0.03	1.00
Primary	Mosaic	SMRegions	1.00	1.00	1.00	1	0	1	inf	inf	inf	1.2E-03	6.8E-03	0.05
Primary	Full	SMRegions	0.31	0.23	0.23	13	9	4	713.1	47.9	6.3	3.5E-10	7.2E-05	0.02
Metastasis	Mosaic	DnDsCV	0.80	0.80	0.80	5	1	4	6992.7	841.8	106.1	1.2E-12	2.8E-09	8.6E-06
Metastasis	Mutect	DnDsCV	0.67	0.67	0.67	6	2	4	3496.2	420.9	53.1	3.6E-12	8.3E-09	2.5E-05
Metastasis	Full	DnDsCV	0.10	0.13	0.18	40	33	7	193.9	30.3	5.6	2.1E-08	1.5E-06	5.5E-04
Primary	Mosaic	DnDsCV	0.50	0.50	0.50	8	4	4	1747.9	210.4	26.5	1.7E-11	3.8E-08	1.1E-04
Primary	Full	DnDsCV	0.20	0.20	0.20	25	20	5	480.3	53.1	6.6	7.2E-12	1.3E-07	1.9E-03
Metastasis	Mosaic	OncodriveFML	1.00	1.00	1.00	4	0	4	inf	inf	inf	1.6E-10	2.2E-08	2.1E-05
Metastasis	Mutect	OncodriveFML	1.00	1.00	1.00	3	0	3	inf	inf	inf	2.1E-06	9.2E-06	4.4E-04
Metastasis	Full	OncodriveFML	0.33	0.33	0.42	12	7	5	303.0	61.6	11.7	9.4E-09	2.4E-06	3.7E-04
Primary	Mosaic	OncodriveFML	0.60	0.60	0.60	5	2	3	2062.0	289.8	36.8	6.1E-09	1.5E-06	5.7E-04
Primary	Full	OncodriveFML	0.36	0.36	0.36	14	9	5	959.5	112.4	14.0	4.6E-13	6.6E-09	1.2E-04
Metastasis	Mosaic	Mutpanning	0.42	0.42	0.42	12	7	5	1329.6	147.1	18.4	1.3E-13	2.4E-09	4.7E-05
Metastasis	Full	Mutpanning	0.21	0.21	0.29	24	17	7	488.7	54.1	10.6	6.8E-12	1.2E-07	2.0E-05
Primary	Mosaic	Mutpanning	0.47	0.40	0.40	15	8	7	2035.9	138.8	17.2	2.7E-19	7.2E-11	1.0E-05
Primary	Full	Mutpanning	0.11	0.10	0.13	71	61	10	336.3	22.9	3.8	1.9E-16	7.3E-08	1.3E-03
Metastasis	Full	HotMaps	0.17	0.25	0.25	12	9	3	99.8	36.7	5.3	3.6E-04	1.8E-04	0.03

Table 2 Supplementary Note 1. Decision made on all genes discovered in the primary and metastasis cohort.

SYMBOL	COHORT	SET	DECISION
ABL2	TCGA	MOSAIC	IN COMPENDIUM
AFF3	TCGA	MOSAIC	IN COMPENDIUM
AFF3	TCGA	FULL	IN COMPENDIUM
APC	HMF	MUTECT	IN COMPENDIUM
ARID2	HMF	MOSAIC	IN COMPENDIUM
ARID2	HMF	MUTECT	IN COMPENDIUM
ASXL1	HMF	FULL	IN COMPENDIUM
ASXL1	HMF	MOSAIC	IN COMPENDIUM
ASXL1	HMF	MUTECT	IN COMPENDIUM
ASXL1	TCGA	MOSAIC	IN COMPENDIUM
ASXL1	TCGA	FULL	IN COMPENDIUM
ATE1	HMF	MOSAIC	IN COMPENDIUM
ATM	HMF	FULL	IN COMPENDIUM
ATM	HMF	MOSAIC	IN COMPENDIUM
ATM	HMF	MUTECT	IN COMPENDIUM
ATM	TCGA	MOSAIC	IN COMPENDIUM
ATM	TCGA	FULL	IN COMPENDIUM
BAX	HMF	FULL	IN COMPENDIUM
CHEK2	HMF	MOSAIC	IN COMPENDIUM
CHEK2	TCGA	MOSAIC	IN COMPENDIUM
CHEK2	TCGA	FULL	IN COMPENDIUM
CUX1	HMF	MUTECT	IN COMPENDIUM
DNM2	HMF	FULL	IN COMPENDIUM
DNMT3A	HMF	FULL	IN COMPENDIUM
DNMT3A	HMF	MOSAIC	IN COMPENDIUM
DNMT3A	HMF	MUTECT	IN COMPENDIUM
DNMT3A	TCGA	MOSAIC	IN COMPENDIUM
DNMT3A	TCGA	FULL	IN COMPENDIUM
ERCC2	TCGA	FULL	IN COMPENDIUM
FOXP1	HMF	MOSAIC	IN COMPENDIUM
GNAS	TCGA	MOSAIC	IN COMPENDIUM
GNAS	TCGA	FULL	IN COMPENDIUM
GNB1	HMF	FULL	IN COMPENDIUM
GNB1	TCGA	MOSAIC	IN COMPENDIUM
GNB1	TCGA	FULL	IN COMPENDIUM
JAK2	HMF	FULL	IN COMPENDIUM
JAK2	HMF	MOSAIC	IN COMPENDIUM
JAK2	TCGA	MOSAIC	IN COMPENDIUM
JAK2	TCGA	FULL	IN COMPENDIUM
KDM5C	HMF	FULL	IN COMPENDIUM
KMT2C	HMF	FULL	IN COMPENDIUM
KMT2C	TCGA	MOSAIC	IN COMPENDIUM
LZTR1	TCGA	MOSAIC	IN COMPENDIUM
MYD88	TCGA	FULL	IN COMPENDIUM
MYO5A	HMF	FULL	IN COMPENDIUM
MYO5A	HMF	MOSAIC	IN COMPENDIUM
NOTCH1	HMF	FULL	IN COMPENDIUM
PABPC1	TCGA	FULL	IN COMPENDIUM
PPFIBP1	TCGA	MOSAIC	IN COMPENDIUM
PPM1D	HMF	FULL	IN COMPENDIUM
PPM1D	HMF	MOSAIC	IN COMPENDIUM
PPM1D	HMF	MUTECT	IN COMPENDIUM
PPM1D	TCGA	MOSAIC	IN COMPENDIUM
PPM1D	TCGA	FULL	IN COMPENDIUM
PTPRD	HMF	MOSAIC	IN COMPENDIUM
RET	HMF	MOSAIC	IN COMPENDIUM
SDHAF2	TCGA	FULL	IN COMPENDIUM
SF3B1	HMF	MOSAIC	IN COMPENDIUM
SF3B1	HMF	MUTECT	IN COMPENDIUM
SMC1A	HMF	MUTECT	IN COMPENDIUM
SRSF2	TCGA	MOSAIC	IN COMPENDIUM
SRSF2	TCGA	FULL	IN COMPENDIUM
STAT3	TCGA	FULL	IN COMPENDIUM
TET2	HMF	FULL	IN COMPENDIUM
TET2	HMF	MOSAIC	IN COMPENDIUM
TET2	HMF	MUTECT	IN COMPENDIUM
TET2	TCGA	MOSAIC	IN COMPENDIUM
TET2	TCGA	FULL	IN COMPENDIUM
TFRC	HMF	FULL	IN COMPENDIUM
TP53	HMF	FULL	IN COMPENDIUM
TP53	HMF	MOSAIC	IN COMPENDIUM
TP53	HMF	MUTECT	IN COMPENDIUM
TP53	TCGA	MOSAIC	IN COMPENDIUM
TP53	TCGA	FULL	IN COMPENDIUM
TP63	HMF	MOSAIC	IN COMPENDIUM
B4GALT5	TCGA	MOSAIC	DISCARDED
CDC42BPA	TCGA	MOSAIC	DISCARDED
CDC42BPA	TCGA	FULL	DISCARDED
FBLN2	HMF	FULL	DISCARDED
FNBP1L	HMF	MOSAIC	DISCARDED
RASAL3	HMF	MOSAIC	DISCARDED
SRCAP	HMF	MOSAIC	DISCARDED
SRCAP	HMF	MUTECT	DISCARDED
ZNF334	HMF	FULL	DISCARDED
ZNF334	HMF	MOSAIC	DISCARDED
ZNF691	HMF	FULL	DISCARDED
ZNF691	HMF	MOSAIC	DISCARDED
ZNF749	HMF	FULL	DISCARDED
ZNF749	HMF	MOSAIC	DISCARDED

Discovery of CH drivers in panel sequencing data

In many clinically oriented initiatives, a subset of all protein-coding genes in solid tumors has been sequenced. In some cases, such as the MSK-IMPACT[43,44], a paired blood sample has also been sequenced with the aim of correctly calling tumor somatic mutations. In two recent studies, the germline variants identified across 24,146 such blood samples have been filtered with those appearing in the tumor sample from the same patient, thus yielding likely blood somatic mutations[12,45].

We hypothesized that the same rationale of discovery of clonal hematopoiesis driver genes presented here could be applied to this cohort (targeted cohort). The genes in the MSK-IMPACT panel have been selected because they are involved in tumor development; most are included in the CGC. In the two aforementioned studies, blood somatic mutations affecting them have been taken as evidence of clonal hematopoiesis. We propose that identifying signals of positive selection in this cohort would fulfill at least two main objectives. First, due to the number of samples included in the cohort, and thus the high statistical power, the discovery would most likely extend the list of drivers of clonal hematopoiesis. Second, given the nature of the genes included in the panel, it would identify cancer driver genes that do not show any evidence to be drivers of clonal hematopoiesis, and thus identified blood mutations may be passenger mutations.

Unlike the discovery described for the metastasis and the primary cohorts, the “panel discovery” of CH drivers is limited to the 468 genes included in the MSK-IMPACT panel. Furthermore, only driver discovery methods that rely on local background models --i.e., capable of computing a background model from the fragment of the genome covered by the panel-- could be employed. We thus applied OncodriveFML[26], OncodriveCLUSTL[36], dNdSscv[35] (without the mutation rate covariates, as described in ref[23]), and SMRegions[37] to the blood somatic mutations identified in these 468 genes across 24,146 samples.

Forty-four drivers of clonal hematopoiesis are identified by at least one method in the targeted cohort discovery (Fig. 4a Supplementary Note 1). Twenty-eight panel CH drivers are identified by more than one method. Twenty-eight of the panel CH driver genes are only identified across the targeted cohort (Fig. 2b of the main paper). Interestingly, 9 genes included in the panel are not identified as CH drivers by the targeted cohort discovery, but are identified either in the primary (4) or in the metastasis (5) cohorts (Fig. 4b Supplementary Note 1). This supports the notion that an extensive effort, including new cohorts is the path to the discovery of the compendium of drivers of clonal hematopoiesis, as has been demonstrated in cancer[19].

One of the main outcomes of this work is an unbiased snapshot of the compendium of clonal hematopoiesis driver genes, presented in Supplementary Table 2 of the main paper and available at www.intogen.org/ch. This compendium is integrated by the genes that exhibit signals of positive selection in their mutational patterns in at least one of the three cohorts analyzed in the paper (whole-exome primary, whole-genome metastasis, and targeted).

a

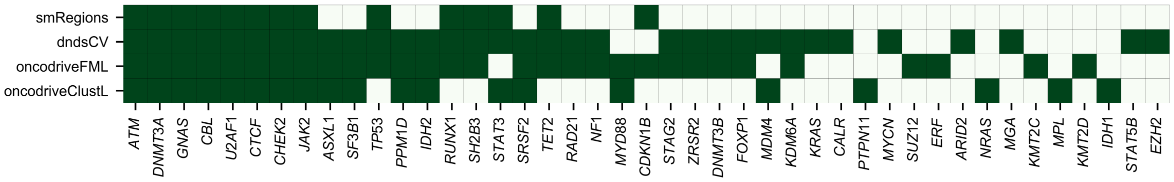
smRegions
dndsCV
oncodriveFML
oncodriveClustL
ATM
DNMT3A
GNAS
CBL
U2AF1
CTCF
CHEK2
JAK2
ASXL1
SF3B1
TP53
PPM1D
IDH2
RUNX1
SH2B3
STAT3
SRSF2
TET2
RAD21
NF1
MYD88
CDKN1B
STAG2
ZRSR2
DNMT3B
FOXP1
MDM4
KDM6A
KRAS
CALR
PTPN11
MYCN
SUZ12
ERF
ARID2
NRAS
MGA
KMT2C
MPL
KMT2D
IDH1
STAT5B
EZH2

b

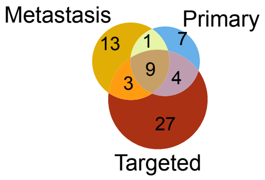
Metastasis
Primary
Targeted
13
1
7
9
3
4
27

Figure 4 Supplementary Note 1. Clonal hematopoiesis drivers identified across targeted sequenced blood samples.
(a) Genes with signals of positive selection (identified by 4 methods as shown in the heatmap) across blood samples probed with the MSK-IMPACT panel (targeted cohort).
(b) Some genes included in the MSK-IMPACT panel only exhibit signals of positive selection in the Primary or Metastasis cohorts (represented by the Venn diagram). This illustrates the importance of carrying out a discovery of CH drivers across cohorts.

Interestingly, although genes in the MSK-IMPACT panels have been selected due to their involvement in tumorigenesis, most of them (424) show no signals of positive selection across these 24,146 blood samples. This includes some genes that appear recurrently mutated in the cohort. For example, *EGFR* or *MED12* well known cancer genes mutated in 58 and 45 blood samples (respectively) in the targeted cohort do not show any signal of positive selection in their mutation patterns. These mutations therefore do not have any support of being drivers of clonal hematopoiesis, and the mutations detected in blood might just be passenger mutations

This observation has important implications for the detection of CH through the identification of somatic mutations in these genes across blood samples. Traditionally, the occurrence of CH in a blood sample is detected either through a mutation affecting a CH driver, or because a number of hematopoiesis mutations are identified in the sample (both approaches are illustrated in Fig. 4g of the main paper). The identification of a mutation in a cancer driver gene which is not a CH driver (in the absence of a critical mass of hematopoiesis mutations detected in the same sample) may lead to a spurious classification of the sample as CH.

Evidences supporting the genes in the compendium
Reassuringly, the discovery of CH driver genes described in the main manuscript identified all well known CH-related genes (Fig. 2 Supplementary Note 1 and of the main manuscript). Moreover, 26 of the newly discovered CH drivers are known to be involved --when mutated-- in the development of myeloid malignancies (Fig. 2 of the main manuscript). For the majority of the remaining genes in the compendium, we found at least one report in the literature supporting their involvement in CH.

We also determined that the age of donors within the primary cohort --that is, those who have not been exposed to cytotoxic treatments-- significantly correlates with the presence of CH mutations in these genes (Supp. Fig. 2b). Interestingly, a study of the relationship between the different tumor types represented in this cohort and the presence of CH showed a lack of significant relationships with most malignancies, with the exception of thymomas, and smaller effects across breast and bladder tumors (Supp. Fig. 2c). This is probably an indication that the donors in this cohort reflect the underlying risk of CH across the general population.

Significance of the compendium of CH drivers
As pointed out in the Discussion of the main paper, the availability of the compendium of clonal hematopoiesis driver genes is significant in at least two regards. First, the compendium of CH drivers will help advance the research on the molecular mechanisms underlying clonal

hematopoiesis faced with different evolutionary constraints (cytotoxic treatments, tobacco carcinogens, etc). Second, knowing the compendium of CH drivers will improve the diagnosis of the condition across human donors, by helping distinguish mutations that more likely drive CH in a donor's blood sample. One can easily imagine that the completion of the compendium will lead to the development of targeted sequencing panels focused on CH drivers. These would guarantee sequencing relevant genes --and, eventually other genomic elements-- at higher depth, thus discovering the condition as early as possible in population screenings.

A second step to take in the direction of identifying CH-driving mutations remains. As the study of tumorigenesis has revealed, not all mutations in CH driver genes will be equally capable of driving clonal hematopoiesis. This is already apparent in the distribution of observed mutations along the sequence of CH drivers presented in Figure 3 of the main paper. The example of mutations affecting *PPM1D* is very eloquent in this sense. In clonal hematopoiesis cases, mutations in this gene tend to be truncating, resulting in the loss of a degron located in the C-terminal portion of the protein, thus leading to its abnormal stabilization which results in decreased levels of the active TP53 protein product[46–49].

It is important to consider that some of the mutations identified in the genes within the compendium --even known CH-related genes-- may not be drivers of CH. For example, while truncating mutations affecting *PPM1D* (see Fig. 4c Supplementary Note 1) which abrogate the degron from the sequence of the protein are known to trigger CH. However, the same has not yet been demonstrated for non-synonymous mutations along the protein sequence. Actually, the same is true in the case of mutations observed in cancer genes in tumorigenesis: at least a fraction of them may be passengers. To assess whether this issue caused an important overestimation of the cases of CH across the metastasis cohort, we repeated the analysis shown in Figure 4g counting only mutations considered to be canonical in CH, according to a recent study[12]. While counting all protein affecting mutations in known CH genes yields 7% of patients with CH in the metastasis cohort (Fig. 4g of the main paper), using the stricter definition in the aforementioned study yields 6.7%. The overestimation is thus, in any case, very small.

References

1. Grossman RL, Heath AP, Ferretti V, et al. Toward a Shared Vision for Cancer Genomic Data. *N. Engl. J. Med.* 2016;375(12):1109–1112.
2. Priestley P, Baber J, Lolkema MP, et al. Pan-cancer whole-genome analyses of metastatic solid tumours. *Nature*. 2019;575(7781):210–216.
3. McKenna A, Hanna M, Banks E, et al. The Genome Analysis Toolkit: A MapReduce framework for analyzing next-generation DNA sequencing data. *Genome Res.* 2010;20(9):1297–1303.
4. Kim S, Scheffler K, Halpern AL, et al. Strelka2: fast and accurate calling of germline and somatic variants. *Nat. Methods*. 2018;15(8):591–594.
5. Morgulis A, Gertz EM, Schäffer AA, Agarwala R. A fast and symmetric DUST implementation to mask low-complexity DNA sequences. *J. Comput. Biol. J. Comput. Mol. Cell Biol.* 2006;13(5):1028–1040.
6. Karimzadeh M, Ernst C, Kundaje A, Hoffman MM. Umap and Bismap: quantifying genome and methylome mappability. *Nucleic Acids Res.* 2018;46(20):e120–e120.
7. Karczewski KJ, Francioli LC, Tiao G, et al. The mutational constraint spectrum quantified from variation in 141,456 humans. *Nature*. 2020;581(7809):434–443.
8. Fujita P a, Rhead B, Zweig AS, et al. The UCSC Genome Browser database: update 2011. *Nucleic Acids Res.* 2011;39(Database issue):D876-82.
9. Sherry ST, Ward MH, Kholodov M, et al. dbSNP: the NCBI database of genetic variation. *Nucleic Acids Res.* 2001;29(1):308–311.
10. Benjamin D, Sato T, Cibulskis K, et al. Calling Somatic SNVs and Indels with Mutect2. *bioRxiv*. 2019;861054.
11. Dou Y, Kwon M, Rodin RE, et al. Accurate detection of mosaic variants in sequencing data without matched controls. *Nat. Biotechnol.* 2020;38(3):314–319.
12. Bolton KL, Ptashkin RN, Gao T, et al. Cancer therapy shapes the fitness landscape of clonal hematopoiesis. *Nat. Genet.* 2020;52(11):1219–1226.
13. Cerami E, Gao J, Dogrusoz U, et al. The cBio Cancer Genomics Portal: An Open Platform for Exploring Multidimensional Cancer Genomics Data. *Cancer Discov.* 2012;2(5):401–404.
14. Alexandrov LB, Nik-Zainal S, Wedge DC, Campbell PJ, Stratton MR. Deciphering Signatures of Mutational Processes Operative in Human Cancer. *Cell Rep.* 2013;3(1):246–259.
15. Pich O, Muiños F, Lolkema MP, et al. The mutational footprints of cancer therapies. *Nat. Genet.* 2019;51(12):1732–1740.
16. Alexandrov LB, Nik-Zainal S, Wedge DC, et al. Signatures of mutational processes in human cancer. *Nature*. 2013;500(7463):415–21.
17. Alexandrov LB, Kim J, Haradhvala NJ, et al. The repertoire of mutational signatures in human cancer. *Nature*. 2020;578(7793):94–101.
18. Osorio FG, Rosendahl Huber A, Oka R, et al. Somatic Mutations Reveal Lineage Relationships and Age-Related Mutagenesis in Human Hematopoiesis. *Cell Rep.* 2018;25(9):2308-2316.e4.
19. Martínez-Jiménez F, Muiños F, Sentís I, et al. A compendium of mutational cancer driver genes. *Nat. Rev. Cancer*. 2020;1–18.
20. Sondka Z, Bamford S, Cole CG, et al. The COSMIC Cancer Gene Census: describing genetic dysfunction across all human cancers. *Nat. Rev. Cancer*. 2018;18(11):696.
21. Fuster José J., Walsh Kenneth. Somatic Mutations and Clonal Hematopoiesis. *Circ. Res.* 2018;122(3):523–532.
22. Zink F, Stacey SN, Norddahl GL, et al. Clonal hematopoiesis, with and without candidate driver mutations, is common in the elderly. *Blood*. 2017;130(6):742–752.
23. Martincorena I, Roshan A, Gerstung M, et al. High burden and pervasive positive selection

of somatic mutations in normal human skin. *Science*. 2015;348(6237):880–886.
24. Buenrostro JD, Corces MR, Lareau CA, et al. Integrated Single-Cell Analysis Maps the Continuous Regulatory Landscape of Human Hematopoietic Differentiation. *Cell*. 2018;173(6):1535-1548.e16.
25. Landrum MJ, Lee JM, Benson M, et al. ClinVar: improving access to variant interpretations and supporting evidence. *Nucleic Acids Res.* 2018;46(D1):D1062–D1067.
26. Mularoni L, Sabarinathan R, Deu-Pons J, et al. OncodriveFML: a general framework to identify coding and non-coding regions with cancer driver mutations. 2016;
27. Combined burden and functional impact tests for cancer driver discovery using DriverPower | Nature Communications.
28. Rheinbay E, Nielsen MM, Abascal F, et al. Analyses of non-coding somatic drivers in 2,658 cancer whole genomes. *Nature*. 2020;578(7793):102–111.
29. Campbell PJ, Getz G, Korbel JO, et al. Pan-cancer analysis of whole genomes. *Nature*. 2020;578(7793):82–93.
30. Kulakovskiy IV, Vorontsov IE, Yevshin IS, et al. HOCOMOCO: towards a complete collection of transcription factor binding models for human and mouse via large-scale ChIP-Seq analysis. *Nucleic Acids Res.* 2018;46(D1):D252–D259.
31. Bernstein BE, Birney E, Dunham I, et al. An integrated encyclopedia of DNA elements in the human genome. *Nature*. 2012;489(7414):57–74.
32. Fishilevich S, Nudel R, Rappaport N, et al. GeneHancer: genome-wide integration of enhancers and target genes in GeneCards. *Database J. Biol. Databases Curation*. 2017;2017:.
33. Grant CE, Bailey TL, Noble WS. FIMO: scanning for occurrences of a given motif. *Bioinformatics*. 2011;27(7):1017–1018.
34. Lopez-Delisle L, Rabbani L, Wolff J, et al. pyGenomeTracks: reproducible plots for multivariate genomic data sets. *Bioinformatics*. .
35. Martincorena I, Raine KM, Gerstung M, et al. Universal Patterns of Selection in Cancer and Somatic Tissues. *Cell*. 2017;171(5):1029-1041.e21.
36. Arnedo-Pac C, Mularoni L, Muiños F, Gonzalez-Perez A, Lopez-Bigas N. OncodriveCLUSTL: a sequence-based clustering method to identify cancer drivers. *Bioinformatics*. 2019;35(22):4788–4790.
37. Martínez-Jiménez F, Muiños F, López-Arribillaga E, Lopez-Bigas N, Gonzalez-Perez A. Systematic analysis of alterations in the ubiquitin proteolysis system reveals its contribution to driver mutations in cancer. *Nat. Cancer*. 2019;
38. Tokheim C, Bhattacharya R, Niknafs N, et al. Exome-scale discovery of hotspot mutation regions in human cancer using 3D protein structure. *Cancer Res.* 2016;76(13):3719–3731.
39. Dietlein F, Weghorn D, Taylor-Weiner A, et al. Identification of cancer driver genes based on nucleotide context. *Nat. Genet.* 2020;52(2):208–218.
40. Weghorn D, Sunyaev S. Bayesian inference of negative and positive selection in human cancers. *Nat. Genet.* 2017;49(12):1785–1788.
41. Sondka Z, Bamford S, Cole CG, et al. The COSMIC Cancer Gene Census: describing genetic dysfunction across all human cancers. *Nat. Rev. Cancer*. 2018;18(11):696–705.
42. Lever J, Zhao EY, Grewal J, Jones MR, Jones SJM. CancerMine: a literature-mined resource for drivers, oncogenes and tumor suppressors in cancer. *Nat. Methods*. 2019;16(6):505–507.
43. Cheng DT, Mitchell T, Zehir A, et al. MSK-IMPACT: A Hybridization Capture-Based Next-Generation Sequencing Clinical Assay for Solid Tumor Molecular Oncology. *J. Mol. Diagn. JMD*. 2015;17(3):251–64.
44. Zehir A, Benayed R, Shah RH, et al. Mutational Landscape of Metastatic Cancer Revealed from Prospective Clinical Sequencing of 10,000 Patients. *Nat. Med.* 2017;23(6):703–713.
45. Coombs CC, Zehir A, Devlin SM, et al. Therapy-Related Clonal Hematopoiesis in Patients

with Non-hematologic Cancers Is Common and Associated with Adverse Clinical Outcomes. *Cell Stem Cell*. 2017;21(3):374-382.e4.
46. Hsu JI, Dayaram T, Tovy A, et al. PPM1D Mutations Drive Clonal Hematopoiesis in Response to Cytotoxic Chemotherapy. *Cell Stem Cell*. 2018;23(5):700-713.e6.
47. Bowman RL, Busque L, Levine RL. Clonal Hematopoiesis and Evolution to Hematopoietic Malignancies. *Cell Stem Cell*. 2018;22(2):157–170.
48. Busque L, Buscarlet M, Mollica L, Levine RL. Concise Review: Age-Related Clonal Hematopoiesis: Stem Cells Tempting the Devil. *STEM CELLS*. 2018;36(9):1287–1294.
49. Challen GA, Goodell MA. Clonal hematopoiesis: mechanisms driving dominance of stem cell clones. *Blood*. 2020;136(14):1590–1598.

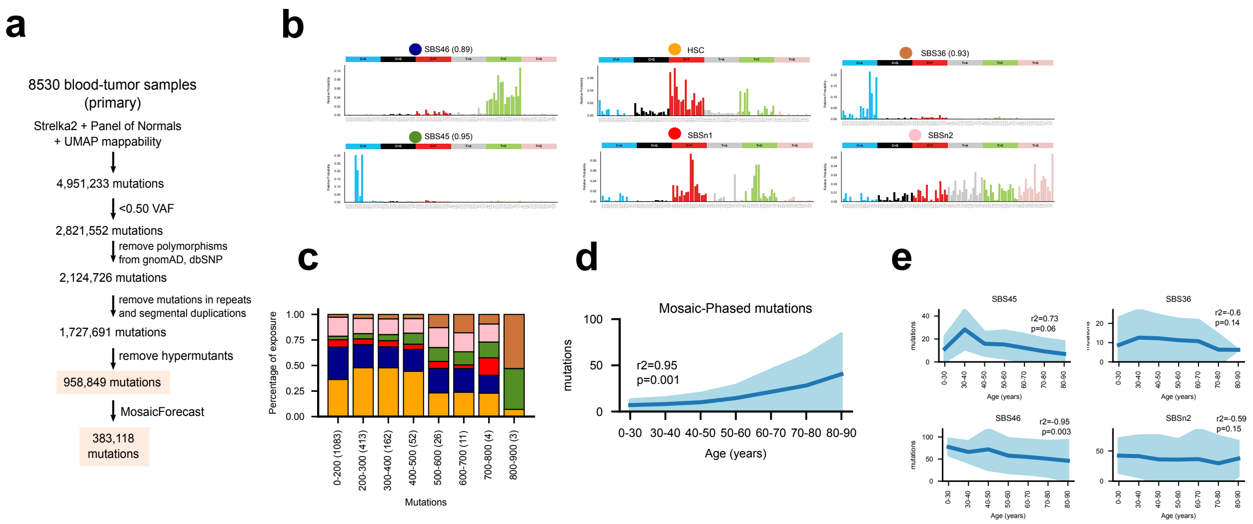

a
8530 blood-tumor samples (primary)
Strelka2 + Panel of Normals + UMAP mappability
4,951,233 mutations
<0.50 VAF
2,821,552 mutations
remove polymorphisms from gnomAD, dbSNP
2,124,726 mutations
remove mutations in repeats and segmental duplications
1,727,691 mutations
remove hypermutants
958,849 mutations
MosaicForecast
383,118 mutations
b
SBS46 (0.89)
HSC
SBS36 (0.93)
SBS45 (0.95)
SBSn1
SBSn2
c
Percentage of exposure
Mutations
0-200 (1083)
200-300 (413)
300-400 (162)
400-500 (52)
500-600 (26)
600-700 (11)
700-800 (4)
800-900 (3)
d
Mosaic-Phased mutations
mutations
r2=0.95
p=0.001
Age (years)
e
SBS45
r2=0.73
p=0.06
SBS36
r2=-0.6
p=0.14
SBS46
r2=-0.95
p=0.003
SBSn2
r2=-0.59
p=0.15
mutations
Age (years)

Supplementary Figure 1. Identification of blood somatic mutations across the primary cohort.

(a) Flowchart of the reverse calling and filtering approach in its application to the primary cohort. In this case only one filter of the set of mutations is applied. Therefore, two sets of somatic mutations are derived from the reverse calling pipeline: the full set and the mosaic set.

(b) Tri-nucleotide profiles of mutational signatures extracted from the somatic mutations in the metastatic cohort (N=3,785 samples).

(c) Distribution of the activity of different mutational signatures active in the metastasis cohort across samples with different burden of somatic mutations.

(d) Significant positive correlation between the number of phased mutations (yielded by the MosaicForecast algorithm) and the age of donors in the metastasis cohort. (The same general trend is shown in Fig. 1e for HSC signature mutations.)

(e) Lack of significant positive correlation between the number of mutations contributed by different mutational signatures (except the corresponding to the HSC signature, depicted in Fig. 1e) and the age of donors in the metastasis cohort.

Source data for panels c, d and e are provided as Source Data files.

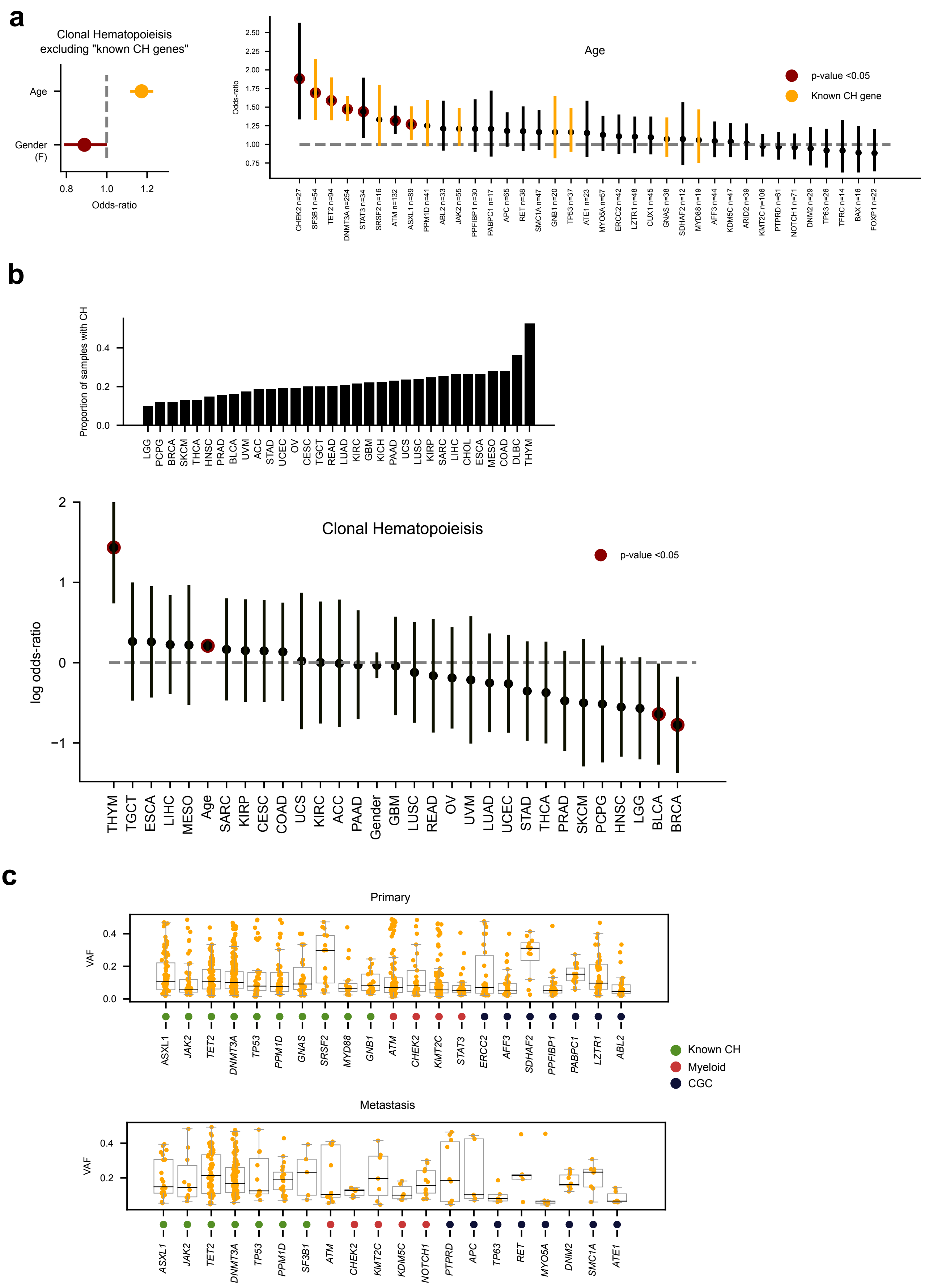
a
Clonal Hematopoieisis excluding "known CH genes"
Age
Gender (F)
Odds-ratio
Age
Odds-ratio
p-value <0.05
Known CH gene
CHEK2 n=27
SF3B1 n=54
TET2 n=94
DNMT3A n=254
STAT3 n=34
SRSF2 n=16
ATM n=132
ASXL1 n=89
PPM1D n=41
ABL2 n=33
JAK2 n=55
PPFIBP1 n=30
PABPC1 n=17
APC n=65
RET n=38
SMC1A n=47
GNB1 n=20
TP53 n=37
ATE1 n=23
MYO5A n=57
ERCC2 n=42
LZTR1 n=48
CUX1 n=45
GNAS n=38
SDHAF2 n=12
MYD88 n=19
AFF3 n=44
KDM5C n=47
ARID2 n=39
KMT2C n=106
PTPRD n=61
NOTCH1 n=71
DNM2 n=29
TP63 n=26
TFRC n=14
BAX n=16
FOXP1 n=22
b
Proportion of samples with CH
Clonal Hematopoieisis
log odds-ratio
p-value <0.05
c
Primary
VAF
Known CH
Myeloid
CGC
Metastasis
VAF

Supplementary Figure 2. The compendium of CH driver genes.

a) Top panel: association between age of patients in the primary cohort and mutation of CH genes in the compendium excluding the list of known CH genes. The significance positive association supports the involvement of the mutations in these novel genes in CH. Bottom panel: association between mutations in individual selected CH drivers (with enough mutations to carry out the regression across the primary cohort) and age. Mutations in most genes are positively associated with age, as expected if they are involved in the development of CH. Significant associations are marked by a black circle surrounding the dot. Yellow: known CH genes. The bars represent the 95% confidence interval of the regression coefficients. The p-values correspond to the results of the logistic regression.

b) Top panel: fraction of donors within each cancer type of the primary cohort with CH mutations. Bottom panel: results of regression of CH on the tumor type (and age) of donors across the primary cohort. The positive effect of age on the development of CH is recapitulated. A significant strong positive relationship between the presence of thymomas (and smaller negative significant effects for breast and bladder tumors) and CH is observed. No significant relationship is appreciable for most tumor types, probably highlighting that the donors in the cohort reflect the underlying risk of CH in the general population. The bars represent the 95% confidence interval of the regression coefficients. The p-values correspond to the results of the logistic regression.

c) Each plot presents the distribution of variant allele frequency (VAF) of mutations in genes identified as CH drivers in the primary (top) and metastasis (bottom) cohorts. Known CH drivers, known myeloid drivers, and novel CH drivers are labeled with different colors (bottom dots), as defined in Figure 2. In the boxplots, the box represents the second and third quartiles, separated by a line indicating the median; the whiskers represent the minimum and maximum of the distribution excluding outliers.

Source data for panels a, b and c are provided as Source Data files.

a

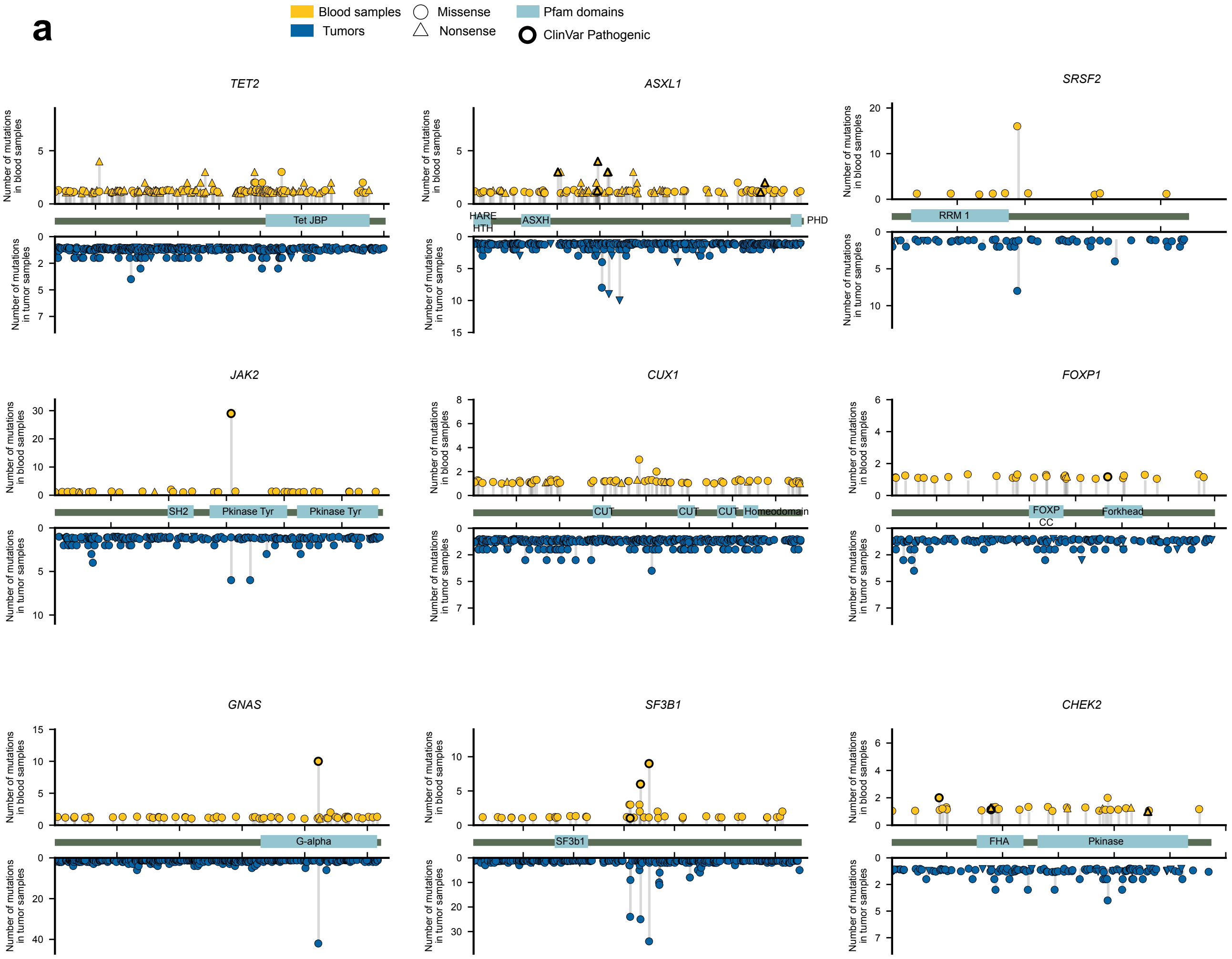
Blood samples
Tumors
Missense
Nonsense
Pfam domains
ClinVar Pathogenic
TET2
ASXL1
SRSF2
JAK2
CUX1
FOXP1
GNAS
SF3B1
CHEK2
Number of mutations in blood samples
Number of mutations in tumor samples
Tet JBP
HARE HTH
ASXH
PHD
RRM 1
SH2
Pkinase Tyr
Pkinase Tyr
CUT
CUT
CUT
Homeodomain
FOXP CC
Forkhead
G-alpha
SF3b1
FHA
Pkinase

Supplementary Figure 3. Distribution of blood and tumor mutations in CH driver genes.
(a) Distribution of blood somatic mutations affecting seven selected genes in the CH drivers compendium (in addition to those presented in Fig. 3b) across donors of the primary and metastasis cohorts (above the horizontal axis) in comparison to those observed in the same genes across 28076 tumors[19] (below the horizontal axis).
Source data are provided as Source Data files.

a

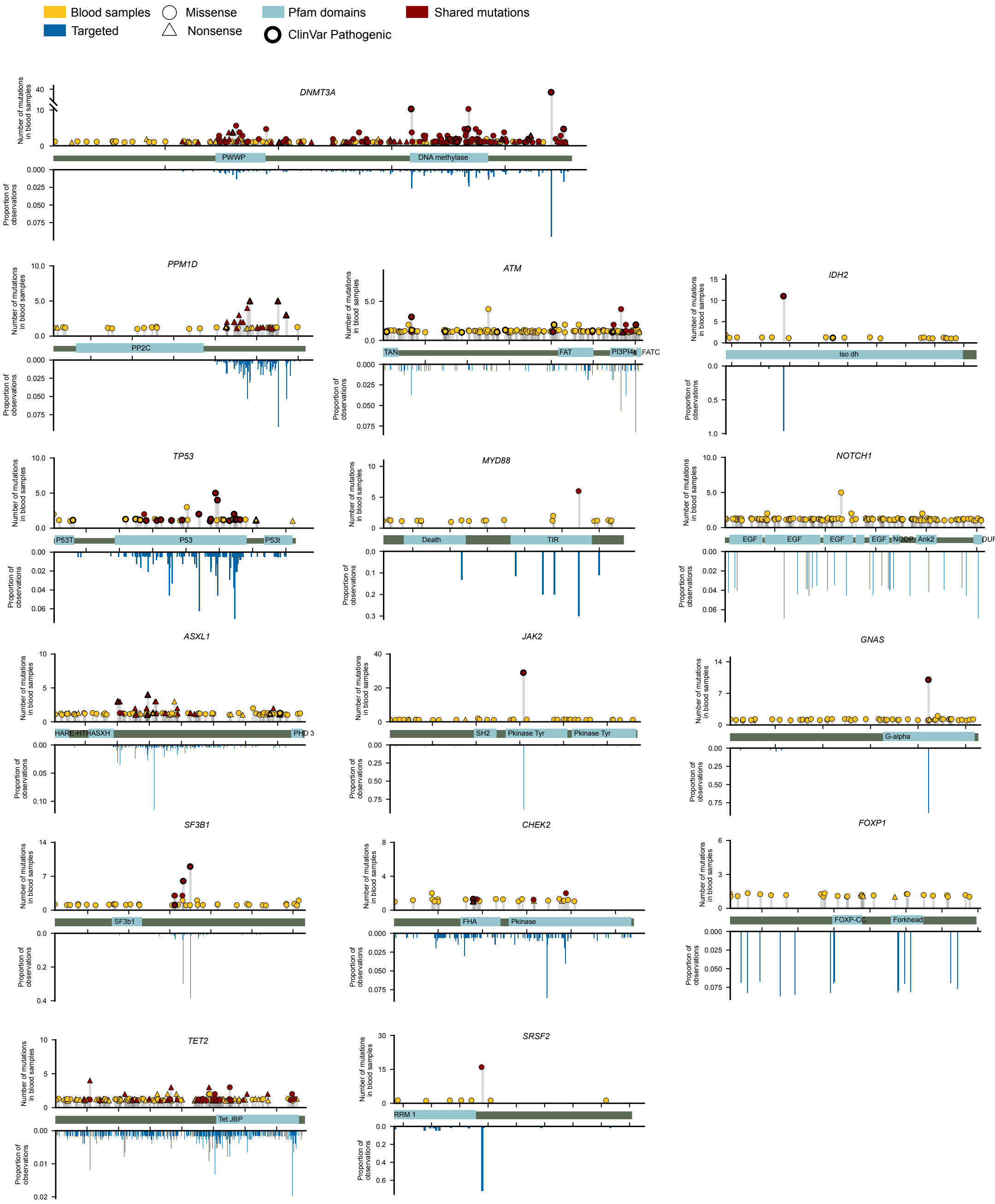

Blood samples
Targeted
Missense
Nonsense
Pfam domains
ClinVar Pathogenic
Shared mutations
Number of mutations in blood samples
Proportion of observations
DNMT3A
PWWP
DNA methylase
PPM1D
PP2C
ATM
TAN
FAT
PI3PI4k
FATC
IDH2
Iso dh
TP53
P53T
P53
P53t
MYD88
Death
TIR
NOTCH1
EGF
NODP
Ank2
DUF3454
ASXL1
HARE-HTH
ASXH
PHD 3
JAK2
SH2
Pkinase Tyr
GNAS
G-alpha
SF3B1
SF3b1
CHEK2
FHA
Pkinase
FOXP1
FOXP-CC
Forkhead
TET2
Tet JBP
SRSF2
RRM 1

b

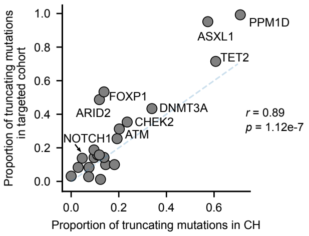

Proportion of truncating mutations in targeted cohort
Proportion of truncating mutations in CH
1.0
0.8
0.6
0.4
0.2
0.0
PPM1D
ASXL1
TET2
FOXP1
ARID2
DNMT3A
CHEK2
ATM
NOTCH1
r = 0.89
p = 1.12e-7

Supplementary Figure 4. Distribution of blood mutations in CH driver genes across different cohorts.

(a) Distribution of blood somatic mutations along the sequence of 15 CH driver genes (presented in Fig. 3b and Supp. Fig. 3) across the primary and metastasis cohorts (above the x-axis) compared to those identified across the targeted cohort (below the x-axis). Proportions with respect to the total number of mutations are shown across the targeted cohort, while the absolute numbers are illustrated for primary and metastasis cohorts.

(b) Fraction of truncating mutations in CH driver genes with 10 or more mutations across the primary and metastatic (x-axis) and the targeted (y-axis) cohorts. The p-value corresponds to the Person's correlation coefficient.

Source data for panels a and b are provided as Source Data files.

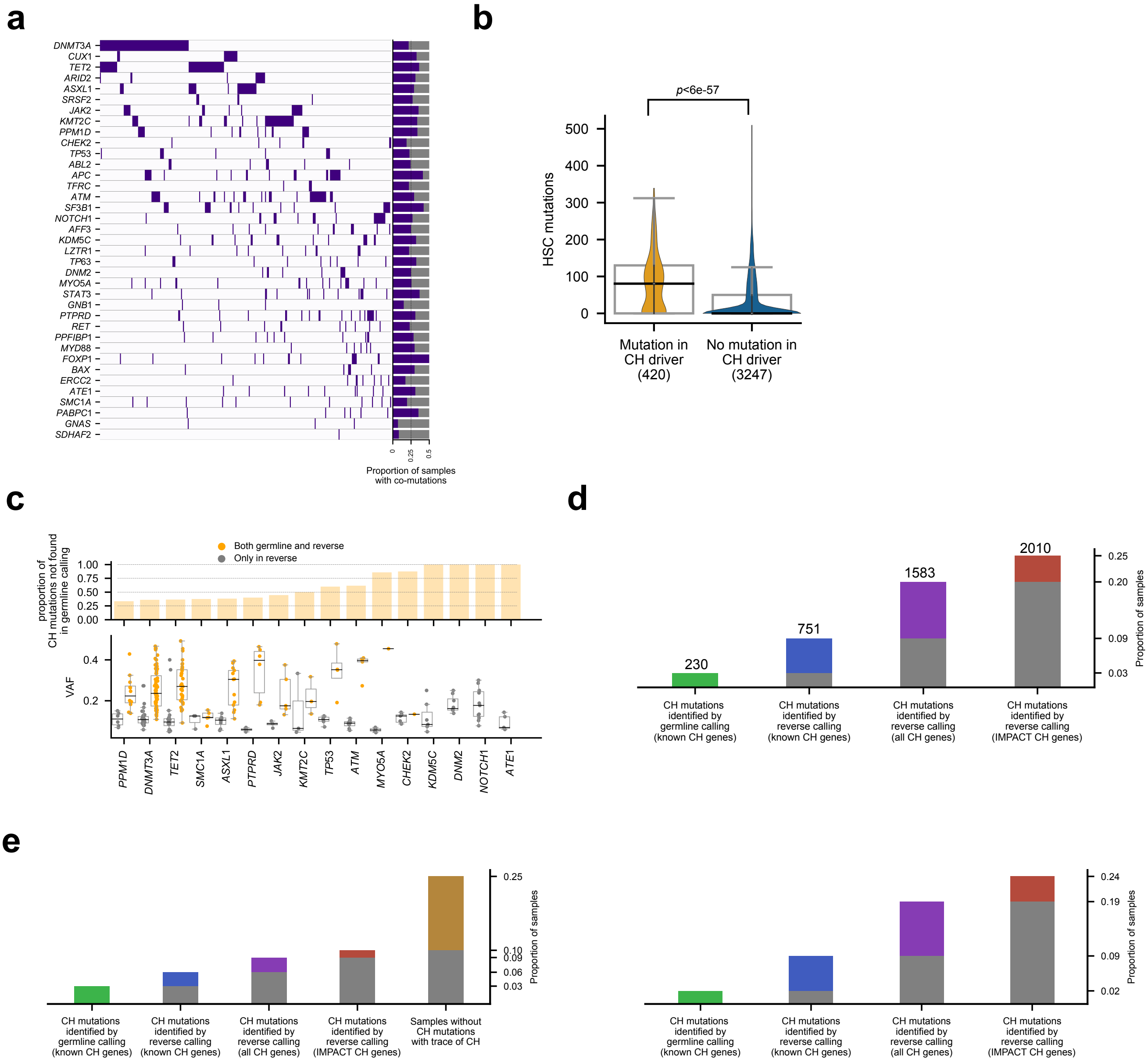

a
DNMT3A
CUX1
TET2
ARID2
ASXL1
SRSF2
JAK2
KMT2C
PPM1D
CHEK2
TP53
ABL2
APC
TFRC
ATM
SF3B1
NOTCH1
AFF3
KDM5C
LZTR1
TP63
DNM2
MYO5A
STAT3
GNB1
PTPRD
RET
PPFIBP1
MYD88
FOXP1
BAX
ERCC2
ATE1
SMC1A
PABPC1
GNAS
SDHAF2
0
0.25
0.5
Proportion of samples with co-mutations
b
p<6e-57
HSC mutations
500
400
300
200
100
0
Mutation in CH driver (420)
No mutation in CH driver (3247)
c
Both germline and reverse
Only in reverse
proportion of CH mutations not found in germline calling
1.00
0.75
0.50
0.25
0.00
VAF
0.4
0.2
PPM1D
DNMT3A
TET2
SMC1A
ASXL1
PTPRD
JAK2
KMT2C
TP53
ATM
MYO5A
CHEK2
KDM5C
DNM2
NOTCH1
ATE1
d
230
751
1583
2010
0.25
0.20
0.09
0.03
Proportion of samples
CH mutations identified by germline calling (known CH genes)
CH mutations identified by reverse calling (known CH genes)
CH mutations identified by reverse calling (all CH genes)
CH mutations identified by reverse calling (IMPACT CH genes)
e
0.25
0.10
0.09
0.06
0.03
Proportion of samples
CH mutations identified by germline calling (known CH genes)
CH mutations identified by reverse calling (known CH genes)
CH mutations identified by reverse calling (all CH genes)
CH mutations identified by reverse calling (IMPACT CH genes)
Samples without CH mutations with trace of CH
0.24
0.19
0.09
0.02
Proportion of samples
CH mutations identified by germline calling (known CH genes)
CH mutations identified by reverse calling (known CH genes)
CH mutations identified by reverse calling (all CH genes)
CH mutations identified by reverse calling (IMPACT CH genes)

Supplementary Figure 5. Detection of clonal hematopoiesis across donors.
(a) Mutation co-occurrence across pairs of CH driver genes.
(b) Distribution of the number of hematopoiesis mutations of donors with mutations in a CH driver gene identified in the primary or the metastasis cohort (N=420) and those with no mutation in CH driver genes (N=3247) across samples from the metastasis cohort. The p-value of the one-tailed Mann-Whitney test is annotated. In the boxplots, the box represents the second and third quartiles, separated by a line indicating the median; the whiskers represent the minimum and maximum of the distribution excluding outliers.
(c) Comparison of the somatic mutations and germline variants identified by the reverse calling approach and a traditional one-sample variant calling across blood samples of donors in the metastasis cohort. Analogous to Figure 1c, but including more known and discovered CH driver genes. The variant allele frequency of mutations affecting *KDM5C* (an X-linked gene) identified in male donors are divided by 2 to correct for their single X dose.
(d) Number of donors (above the bars) in the primary cohort with clonal hematopoiesis recognizable through different criteria. Similar to Figure 4g, but only with the first four sets of donors. The fifth set is not available due to the extreme difficulty of extracting a mutational signature with the low number of mutations provided by whole-exome sequencing.
(e) Number of donors in the metastasis (left) and primary (right) cohorts with clonal hematopoiesis recognizable through different criteria. Similar to Figure 4g and Supplementary Figure 5d, but restricting blood somatic mutations to those with VAF<=0.4. The numbers are almost identical, showing that there are very few cases in which mutations with VAF ranging between 0.4 and 0.5 affect genes of the compendium.
Source data for panels a, b, c, d and e are provided as Source Data files.

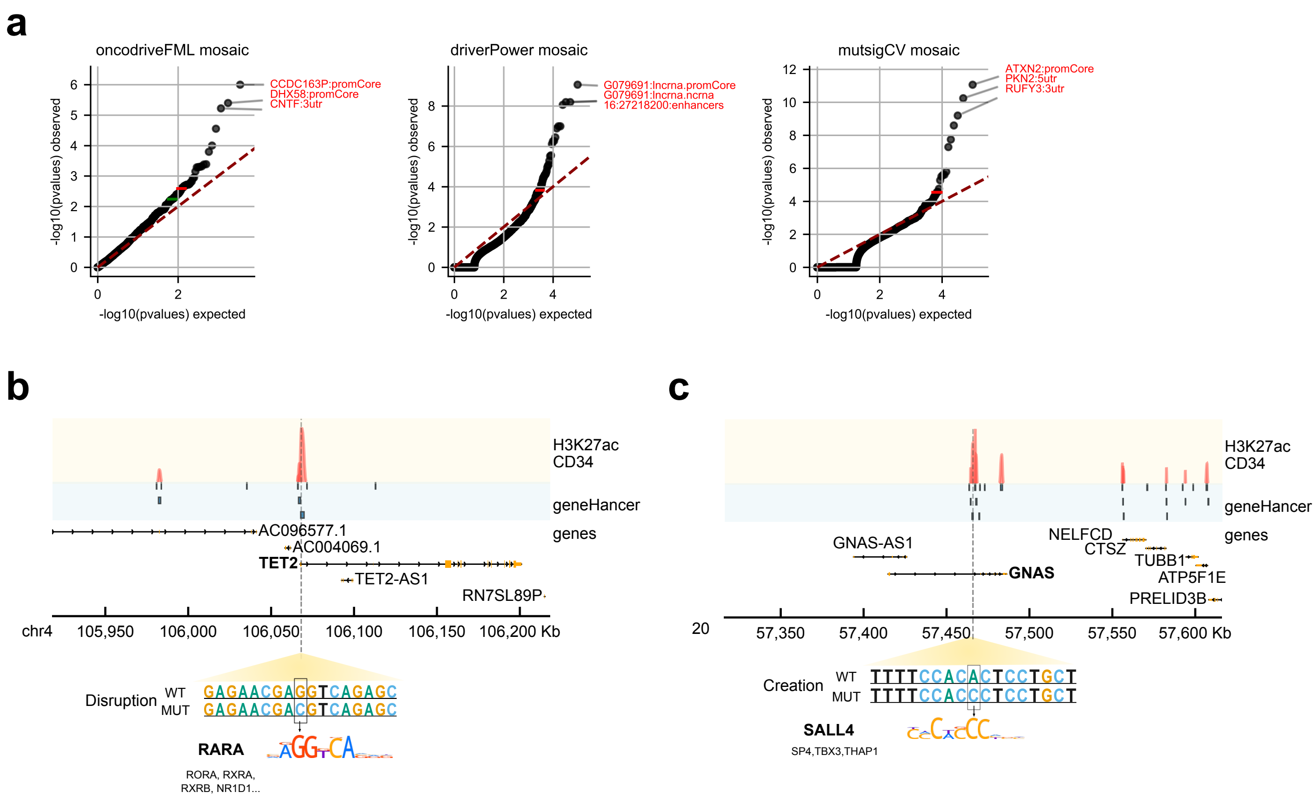
a
oncodriveFML mosaic
CCDC163P:promCore
DHX58:promCore
CNTF:3utr
driverPower mosaic
G079691:lncrna.promCore
G079691:lncrna.ncrna
16:27218200:enhancers
mutsigCV mosaic
ATXN2:promCore
PKN2:5utr
RUFY3:3utr
-log10(pvalues) observed
-log10(pvalues) expected
b
H3K27ac CD34
geneHancer
genes
AC096577.1
AC004069.1
TET2
TET2-AS1
RN7SL89P
chr4
105,950
106,000
106,050
106,100
106,150
106,200 Kb
Disruption
WT GAGAACGAGGTCAGAGC
MUT GAGAACGACGTCAGAGC
RARA
RORA, RXRA, RXRB, NR1D1...
c
H3K27ac CD34
geneHancer
genes
GNAS-AS1
NELFCD
CTSZ
TUBB1
ATP5F1E
PRELID3B
GNAS
20
57,350
57,400
57,450
57,500
57,550
57,600 Kb
Creation
WT TTTTCCACACTCCTGCT
MUT TTTTCCACCCTCCTGCT
SALL4
SP4,TBX3,THAP1

Supplementary Figure 6. Non-coding mutations in clonal hematopoiesis.

(a) Quantile-quantile plots presenting the observed and expected distribution of p-values resulting from the analysis of blood somatic mutations overlapping non-coding genomic elements across the metastasis cohort with three state-of-the-art non-coding driver discovery methods. The names of the most significant non-coding genomic elements are annotated in red in the plot. The empirical or non-parametric tests implemented by each of the methods are described in their respective articles.

(b) Example of a mutation that potentially disrupts the binding site of an expressed transcription factor within an enhancer element in the genome of a donor in the metastasis cohort. Enhancers are obtained from a manually curated database (geneHancer) and bear marks of transcriptional activity (i.e, active enhancers) in cells with phenotype close to HSC. Other possible affected transcription factors are also labeled.

(c) Example of a mutation that potentially creates a binding site for an expressed transcription factor in one enhancer element. Other possible affected transcription factors are also labeled.

Source data are provided as Source Data files.
