# Supplementary material for: Discovering the drivers of clonal hematopoiesis
Source: Nat Commun. 2022 Jul 23;13:4267. doi: 10.1038/s41467-022-31878-0 (PMC9308779; doi:10.1038/s41467-022-31878-0)
Supplement: Supplementary file 2 — Description of Additional Supplementary File [file 41467_2022_31878_MOESM2_ESM.pdf]

Description of Additional Supplementary File

Discovering the drivers of clonal hematopoiesis

Oriol Pich[1], Iker Reyes-Salazar[1], Abel Gonzalez-Perez[1,2,^], Nuria Lopez-Bigas[1,2,3,^]

1. Institute for Research in Biomedicine (IRB Barcelona), The Barcelona Institute of Science and Technology, Baldiri Reixac, 10, 08028 Barcelona, Spain.
2. Research Program on Biomedical Informatics, Universitat Pompeu Fabra, Barcelona, Catalonia, Spain.
3. Institució Catalana de Recerca i Estudis Avançats (ICREA), Barcelona, Spain

^Corresponding authors
Abel Gonzalez-Perez: abel.gonzalez@irbbarcelona.org
Nuria Lopez-Bigas: nuria.lopez@irbbarcelona.org

Supplementary Data file 1. Mutational profiles of signatures active across blood samples in the metastasis cohort.
Supplementary Data file 2. Compendium of clonal hematopoiesis driver genes.
Supplementary Data file 3. Non-coding elements significant by the analysis of three driver discovery methods.
Supplementary Data file 4. Examples of mutations potentially affecting transcription factor binding sites in enhancer elements. P-values associated with the score of Transcription Factor binding to wild-type sequences within regulatory elements were computed using FIMO (see main Methods.)
