# Supplementary material for: Discovering the drivers of clonal hematopoiesis
Source: Nat Commun. 2022 Jul 23;13:4267. doi: 10.1038/s41467-022-31878-0 (PMC9308779; doi:10.1038/s41467-022-31878-0)
Supplement: Supplementary file 7 — Reporting Summary [file 41467_2022_31878_MOESM7_ESM.pdf]

nature portfolio

Corresponding author(s): Nuria Lopez-Bigas, Abel Gonzalez-Perez

Last updated by author(s): Apr 29, 2022

Reporting Summary

Nature Portfolio wishes to improve the reproducibility of the work that we publish. This form provides structure for consistency and transparency in reporting. For further information on Nature Portfolio policies, see our Editorial Policies and the Editorial Policy Checklist.

Statistics

For all statistical analyses, confirm that the following items are present in the figure legend, table legend, main text, or Methods section.

n/a	Confirmed	
☐	☒	The exact sample size (*n*) for each experimental group/condition, given as a discrete number and unit of measurement
☒	☐	A statement on whether measurements were taken from distinct samples or whether the same sample was measured repeatedly
☐	☒	The statistical test(s) used AND whether they are one- or two-sided<br>*Only common tests should be described solely by name; describe more complex techniques in the Methods section.*
☐	☒	A description of all covariates tested
☐	☒	A description of any assumptions or corrections, such as tests of normality and adjustment for multiple comparisons
☐	☒	A full description of the statistical parameters including central tendency (e.g. means) or other basic estimates (e.g. regression coefficient) AND variation (e.g. standard deviation) or associated estimates of uncertainty (e.g. confidence intervals)
☐	☒	For null hypothesis testing, the test statistic (e.g. *F*, *t*, *r*) with confidence intervals, effect sizes, degrees of freedom and *P* value noted<br>*Give P values as exact values whenever suitable.*
☒	☐	For Bayesian analysis, information on the choice of priors and Markov chain Monte Carlo settings
☒	☐	For hierarchical and complex designs, identification of the appropriate level for tests and full reporting of outcomes
☒	☐	Estimates of effect sizes (e.g. Cohen's *d*, Pearson's *r*), indicating how they were calculated

Our web collection on statistics for biologists contains articles on many of the points above.

Software and code

Policy information about availability of computer code

Data collection

-The sequences of solid tumors and their paired blood samples (BAM files), as well as the germline variants across donors were obtained from the Genomic Data Commons (GDC; https://portal.gdc.cancer.gov65) portal upon dbGAP request (phs000178.v11.p8 dataset; https://www.ncbi.nlm.nih.gov/projects/gap/cgi-bin/study.cgi?study_id=phs000178.v11.p8) for the primary cohort (N=8,530) and from the Hartwig Medical Foundation (HMF; https://www.hartwigmedicalfoundation.nl 29) repository, upon request to HMF for the metastatic cohort (N=3,785).
-Blood somatic mutations identified across 24,146 targeted-sequenced samples were directly obtained from cBioportal (https://www.cbioportal.org/study/summary?id=msk_ch_2020).
-Whole-genome somatic variants of 23 blood samples from healthy donors of different ages were obtained from Osorio et al (Osorio FG, Rosendahl Huber A, Oka R, et al. Somatic Mutations Reveal Lineage Relationships and Age-Related Mutagenesis in Human Hematopoiesis. Cell Rep. 2018;25(9):2308-2316.e4.)
-Mutations observed across hematopoietic malignancies in CH genes were obtained from IntOGen (intogen.org)
-ClinVar pathogenic variants were obtained from ClinVar ftp.
-Human polymorphisms were obtained from gnomAD v.2.1 and dbSNP151.
-RNA levels of CH genes in bone marrow CD34+ cells was obtained from GEO (GSE96811).
-H3K27ac ChIP data for CD34+ cells was obtained from ENCODE.

Data analysis	-In the reverse calling of blood somatic variants, Strelka2 v2.9 and Mutect2 (via GATK command v4.1) were used.<br>-MosaicForecast (https://github.com/parklab/MosaicForecast) v.0.0.1<br>-Mutational signatures were extracted using the SigProfilerJulia (bitbucket.org/bbglab/sigprofilerJulia) in-house implementation of the SigProfiler algorithm.<br>-The discovery of genes with signals of positive selection was carried out using the IntOGen pipeline release 2020-02-01 (intogen.org)<br>-OncodriveFML v2.4, DriverPower v1.0.2 and MutSigCV_NC were used to identify signals of positive selection in non-coding genomic elements.

For manuscripts utilizing custom algorithms or software that are central to the research but not yet described in published literature, software must be made available to editors and reviewers. We strongly encourage code deposition in a community repository (e.g. GitHub). See the Nature Portfolio guidelines for submitting code & software for further information.

Data

Policy information about availability of data

All manuscripts must include a data availability statement. This statement should provide the following information, where applicable:

- Accession codes, unique identifiers, or web links for publicly available datasets
- A description of any restrictions on data availability
- For clinical datasets or third party data, please ensure that the statement adheres to our policy

The sequencing data to carry out the reverse calling of blood somatic mutations (and germline variants across donors) is available via dbGaP (TCGA; phs000178.v11.p8; [https://www.ncbi.nlm.nih.gov/projects/gap/cgi-bin/study.cgi?study_id=phs000178.v11.p8]) and HMF (https://hartwigmedical.github.io/documentation/data-access-request-application.html, version DR110). Access to these protected data must be requested from TCGA and HMF. The procedure and conditions to access these datasets are detailed in the sites referenced above. Gene expression in bone marrow CD34+ cells are available at The Gene Expression Omnibus (GSE96811; [https://www.ncbi.nlm.nih.gov/geo/query/acc.cgi?acc=GSE96811]). H3K27ac ChIP data for CD34+ samples are available from ENCODE ([https://www.encodeproject.org/experiments/ENCSR891KSP/]). Mutations in CH drivers across hematopoietic malignancies are available from IntOGen (intogen.org). Disease-related variants are available from ClinVar (https://ftp.ncbi.nlm.nih.gov/pub/clinvar/).

We have prepared flat files containing the set of blood somatic mutations identified in both datasets and have made them available through HMF and dbGaP following the same procedure to access the original datasets. HMF blood somatic mutations are available as part of the data access request to HMF (see above). TCGA blood somatic mutations are available through dbGaP (phs002867; [https://www.ncbi.nlm.nih.gov/projects/gap/cgi-bin/study.cgi?study_id=phs002867.v1.p1]) to researchers who have obtained permission to access protected TCGA data. Panel-sequenced data from the IMPACT targeted cohort is available through cBioPortal ([https://www.cbioportal.org/study/summary?id=msk_ch_2020]). The compendium of CH drivers is available via www.intogen.org/ch. Other datasets employed in specific analyses are described in prior sections of these Methods and in README files within the code repository. Source data are provided with this paper.

Field-specific reporting

Please select the one below that is the best fit for your research. If you are not sure, read the appropriate sections before making your selection.

☒ Life sciences ☐ Behavioural & social sciences ☐ Ecological, evolutionary & environmental sciences

For a reference copy of the document with all sections, see nature.com/documents/nr-reporting-summary-flat.pdf

Life sciences study design

All studies must disclose on these points even when the disclosure is negative.

Sample size	All available samples from solid tumors in the metastasis (3,785), primary (8,530) and targeted (24,146) cohorts.
Data exclusions	Tumors of hematopoietic origin in which a full clonal expansion has occurred from a hematopoietic stem cell, precluding the presence of clonal hematopoiesis, an incomplete clonal expansion.
Replication	Analysis were replicated across the three aforementioned cohorts
Randomization	All samples were included in the reverse calling and posterior analyses, precluding randomization
Blinding	All analyses carried out (logistic regressions, group comparisons, etc) required identification of the samples in groups

Reporting for specific materials, systems and methods

We require information from authors about some types of materials, experimental systems and methods used in many studies. Here, indicate whether each material, system or method listed is relevant to your study. If you are not sure if a list item applies to your research, read the appropriate section before selecting a response.

Materials & experimental systems

n/a	Involved in the study
☒	☐ Antibodies
☒	☐ Eukaryotic cell lines
☒	☐ Palaeontology and archaeology
☒	☐ Animals and other organisms
☒	☐ Human research participants
☒	☐ Clinical data
☒	☐ Dual use research of concern

Methods

n/a	Involved in the study
☒	☐ ChIP-seq
☒	☐ Flow cytometry
☒	☐ MRI-based neuroimaging
